# 

**Published:** 1848-09

**Authors:** 


Notices of new publications, and other editorial articles are crowded out of this number. A Review by Prof. Lee, received too late for this number, will appear in our next. The reader is requested to make the following corrections in the article by Dr. C. Green in our last number, on the preparation of chloroform,—in the last paragraph, for ℥v read ʒv. In the last paragraph but one, second line, for *backwards*, read *forwards*.

